# Predictors and prognosis of PCI-related myocardial injury in chronic total occlusion

**DOI:** 10.1186/s12872-022-02887-0

**Published:** 2022-10-29

**Authors:** Tianzhong Kong, Xintong Dai, Bo Luan, Xiaojiao Zhang, Aijie Hou, Yong Wang

**Affiliations:** 1 Dalian Medical University, Lushunkou District, No. 9, West section of Lushun South Road, 116041 Dalian, China; 2 Department of cardiology, The People’s Hospital of Liaonig Province, No.33, Wenyi road, Shenhe District, 110000 Shenyang City, Liaoning Province China; 3 Present Address: Department of Cardiology, Shenzhen Luohu Hospital Group Luohu People’s Hospital (The Third Affiliated Hospital of Shenzhen University), shenzhen City, China

**Keywords:** Chronic total occlusion, Periprocedural myocardial injury, Percutaneous coronary intervention, Coronary artery disease

## Abstract

**Background:**

Periprocedural myocardial injury (PMI) is associated with major adverse cardiovascular events (MACE) after percutaneous coronary intervention (PCI). However, the incidence predictors and prognosis of PMI in chronic total occlusion (CTO) undergoing PCI remains unclear.

**Method:**

To evaluate the predictors and prognostic impact of PMI following PCI in patients with CTO. We consecutively enrolled 132 individuals and 8 of whom with procedural failure were excluded in this study. Thus, a total of 124 CTO patients successfully received PCI were included in this study. And patients were divided into the PMI group (n = 42) and the non-PMI group (n = 82) according to their c-TnI levels measured after procedure. The baseline and angiographic characteristics of the two groups were compared. The predictors of PMI and the correlation between PMI and MACE were investigated.

**Results:**

Overall, PMI occurred in 42 patients (33.9%). Comparing with control group, PMI group had more diabetes (54.8% vs. 31.7%,P = 0.013) and dyslipidemia (54.8% vs. 13.4%, P＜0.001). Also, there were significant differences between the two groups in left ventricular ejection fraction(43.2 ± 7.2 vs 47.2 ± 8.0, P = 0.027), prior myocardial infarction(54.8%vs43.1%, P = 0.020), prior PCI(57.1% vs 22.0%, P＜0.001) and prior CABG(14.3% vs 2.4%, P = 0.011). Also, patients with PMI had more calcified lesions (52.4% vs 24.4%, P = 0.002) and were more likely to have multivessel disease (71.4% vs 35.4%, P＜0.001). In addition, patients in the PMI group had higher J-CTO scores (3.3 ± 1.0 vs 1.9 ± 0.5, P＜0.001) and were more likely to have wire-crossing difficulties (64.3% vs 37.8%, P = 0.005), require more use of retrograde approach (38.1% vs 7.3%, P＜0.001) and have more procedural complications (19.0% vs 2.4%, P = 0.003). In the multivariate analysis, multivessel artery disease (odd ratio [OR], 4.347;95% confidence interval [CI], 1.601– 11.809;P  = 0.004), retrograde approach (OR, 4.036; 95%CI, 1.162– 14.020;P  = 0.028) and the presence of procedural complications (OR, 16.480;95%CI, 2.515-107.987;P = 0.003) were predictors of PMI.

**Conclusion:**

The incidence of PMI in CTO patients after PCI was 33.9%. Multivessel artery disease, retrograde approach, and the presence of procedural complications were predictors of PMI after CTO-PCI. Patients who develop PMI tend to have a poorer clinical prognosis and more MACE than those who do not develop PMI.

## Introduction

Chronic total occlusion (CTO) is present in approximately 15–20% of patients who undergo coronary angiography [1]. Compared with interventions for non-CTO lesions, CTO–percutaneous coronary intervention (CTO-PCI) is more difficult to perform and may encounter a greater possibility of periprocedural complications [2]. Periprocedural myocardial injury (PMI) is a common complication of PCI. The incidence of PMI varies from 15 to 40% depending on the definition and the cardiac markers used [3]. The occurrence of PMI may be related to side-branch occlusion and distal thromboembolism generated during the CTO-PCI [4, 5]. Studies have shown that, in patients treated with PCI, the presence of PMI may lead to a higher risk of mortality and MACE [6]. However, few studies have reported the predictors and clinical significance of PMI after CTO-PCI. This study used the fourth universal definition of myocardial infarction (type 4a myocardial infraction), in which cardiac troponin (cTn) is elevated more than 5 times the upper limit of the 99% reference value after PCI [7], to define PMI and examine the determinants of PMI occurrence in patients undergoing CTO-PCI as well as the occurrence of MACE in hospital and during one month follow up.

PMI Periprocedural myocardial injury, PCI percutaneous coronary intervention, CTO, chronic total occlusion, cTnI cardiac troponin-I,

## Method

### Study design and population

This study prospectively enrolled 132 consecutive patients who underwent CTO-PCI. Eight patients were excluded from the analysis because PCI failure and serious procedural complications. (e.g., coronary perforation, requiring urgent surgical intervention, acute cardiac tamponade) All procedures were performed by qualified and experienced physician. All patients underwent preoperative preparation and had no procedural contraindications. Patients who experienced procedural complications were treated promptly in the catheter room. For patients with dissection/hematomas, we timely implant stents to prevent dissection expansion. For patients with perforation, on the one hand, we expand blood volume and increase blood pressure. On the other hand, we use pericardiocentesis or emergency surgical pericardiotomy for treatment.

**Fig. 1:**
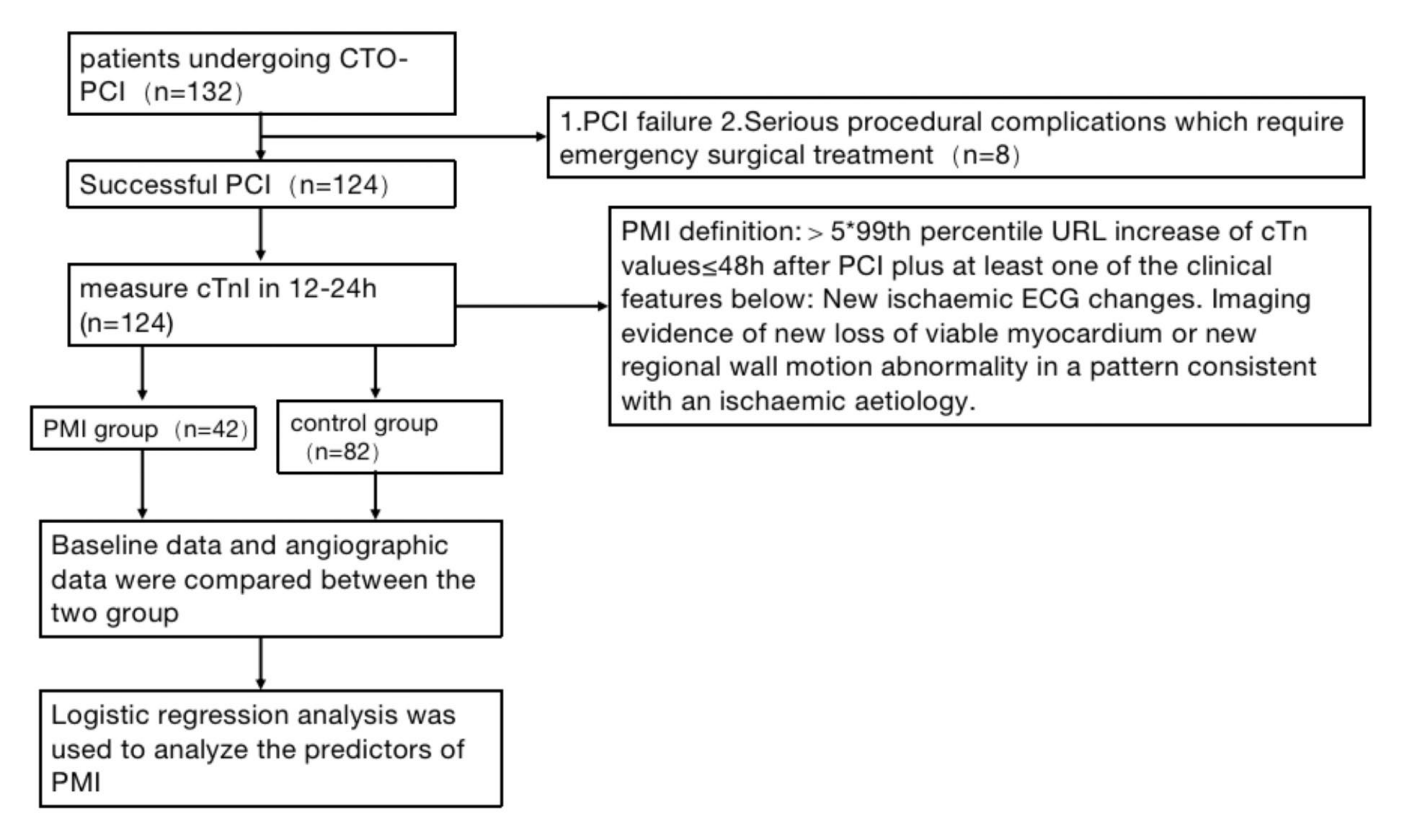
Study flow chart

Patients who developed postprocedural MACE were also treated in the hospital in a timely manner. Relevant baseline information was collected from the patients, including age, sex, body mass index, LVEF, creatinine level, C-reactive protein (CRP), history of previous disease, and history of previous procedure. Procedural imaging-related data were collected, including lesion site, presence of calcification, lesion length, and Japan-CTO score (J-CTO). Troponin level testing, electrocardiography, and echocardiography were performed before and 12 h after procedure, and the patients were divided into two groups according to the presence or absence of PMI. The MACE within one month after procedure was recorded to analyze the predictors of PMI (Fig. 1). This study was approved by the Institutional Ethics Committee and informed consent was obtained from all individuals involved in the study. This study was conducted in accordance with the principles of the Declaration of Helsinki.

### Definitions and study endpoints

Coronary CTO was defined as total occlusion with Thrombolysis in Myocardial Infarction (TIMI) grade flow 0 with at least a 3-month duration [8]. The time of occlusion was determined by the patient’s first angina symptom or previous angiography. Procedural success was defined as residual stenosis less than 30% with distal blood flow of TIMI 3 grade. PMI was defined as type 4a myocardial infarction which cTnI is elevated more than 5 times the upper limit of the 99% reference value after PCI. There were also new angina symptoms, ischemic changes in electrocardiogram, and new ventricular wall movement abnormalities on imaging [7]. Procedural complications were defined as target or side-branch dissection, hematoma, and perforation without surgical intervention. The endpoints were MACE occurred in hospital and during one month follow up including all-cause death, revascularization of target vessels, acute heart failure and acute myocardial infarction.

### Statistical analysis

Statistical package for Social Sciences (SPSS) 26.0 software was used for data analysis. Normally distributed continuous data were expressed as mean ± standard deviation and those not normally distributed were shown as median (min-max). To compare continuous variables, the Student t-test or Mann-Whitney U test were used. Categorical variables were expressed in frequency (percentage), and were compared using Chi-square test or Fisher’s exact test as appropriate according to sample size. Logistic regression analysis was used to analyze the predictors of PMI. P values < 0.05 was defined as statistically significant difference.

## Results

### Baseline characteristics

A total of 124 patients (96.1%) undergoing CTO-PCI were included in the study, of whom 42 (33.9%) had confirmed PMI and 82 (66.1%) did not have PMI. Patients with PMI had a higher incidence of diabetes (54.8% vs. 31.7%, P = 0.013) and dyslipidemia (54.8% vs. 13.4%, P＜0.001)than control group. In addition, there were significant differences between the two groups in LVEF (43.2 ±  7.2 vs 47.2  ±  8.0, P = 0.027), prior myocardial infarction(54.8% vs 43.1%, P = 0.020), prior PCI (57.1%vs22.0%, P＜0.001) and prior CABG (14.3% vs 2.4%, P = 0.011) (Table 1).

**Table 1:** *Clinical characteristics of the two groups* Number(%) M(P25,P75), mean ± SD

	PMI group(n = 42)	Control group (n = 82)	P value
Age＞65years	25(59.5)	47(57.3)	0.814
Female gender	16(38.1)	28(43.1)	0.664
BMI(Kg/m^2^)	25.3 ± 3.6	24.5 ± 3.1	0.322
BMI＞28Kg/m^2^	9(21.4)	14(17.1)	0.555
Smoke,current	21(50.0)	45(54.9)	0.606
Hypertension	25(59.5)	42(51.2)	0.380
Diabetes	23(54.8)	26(31.7)	0.013
Hyperlipidaemia	23(54.8)	11(13.4)	＜0.001
Creatinine(µmol/l)	78.6 ± 39.2	77.6 ± 23.4	0.744
CRP rise	18(42.9)	27(32.9)	0.276
LVEF %	43.2 ± 7.2	47.2 ± 8.0	0.027
Anemia	13(31.0)	17(20.7)	0.208
Prior myocardial infarction	23(54.8)	28(34.1)	0.020
Prior stroke	18(42.9)	34(41.5)	0.882
Prior PCI	24(57.1)	18(22.0)	＜0.001
Prior CABG	6(14.3)	2(2.4%)	0.011

Data presented as mean ± standard deviation or number (%); Bold values highlight the p values under statistical significance (< 0.05); PCI, percutaneous coronary intervention; CABG, coronary artery bypass graft; PMI, periprocedural myocardial injury; LVEF, left ventricular ejection fraction; BMI, body mass index; CRP, C-reactive protein

### Angiographic and procedural characteristics

There was no significant difference in CTO target vessels, lesion length, the number of stents and the volume of contrast used. However, patients with PMI had more calcified lesions (52.4% vs 24.4%, P = 0.002) and were more likely to have multivessel artery disease (71.4% vs 35.4%, P＜0.001). Patients in the PMI group had higher J-CTO scores (3.3 ± 1.0 vs 1.9 ± 0.5, P＜0.001) and were more likely to have a guidewire crossing time more than 30 min (64.3% vs 37.8%, P = 0.005). In addition, patients with PMI had a higher prevalence of retrograde approach (38.1% vs 7.3%, P＜0.001) and also a higher procedural complications (19.0% vs 2.4%, P = 0.003) (Tables 2 and 3).

**Table 2:** *Baseline angiographic characteristics* Number(%) M(P25,P75), mean ± D

	PMI group(n = 42)	Control group (n = 82)	P value
Multivessel disease^a^	30(71.4)	29(35.4)	p＜0.001
CTO target vessel			0.639
LAD	16(38.1)	25(30.5)	
LCX	8(19.0)	15(18.3)	
RCA	18(42.9)	42(51.2)	
CTO length＞15 mm	33(78.6)	61(74.4)	0.607
Calcification	22(52.4)	20(24.4)	0.002
J-CTO score (mean)	3.3 ± 1.0	1.9 ± 0.5	P＜0.001
J-CTO score classification			0.004
0	0(0)	8(9.8)	
1	9(21.4)	32(39.0)	
2	15(35.7)	27(32.9)	
≥ 3	18(42.9)	15(18.3)	
Rentrop grade ≥ 2	29(69.0)	49(59.8)	0.311

Data presented as mean±standard deviation or number (%); Bold values highlight the p values under statistical significance (<0.05); CTO, chronic total occlusion; J-CTO score, Japan CTO score; LAD, left anterior descending artery; LCX, left circumflex artery; RCA, right coronary artery

^a^ Defined as＞50% stenosis in 2 or more major coronary arteries

**Table 3:** *Procedural characteristics and complications.* Number(%) M(P25,P75), mean ± SD

	PMI group(n = 42)	Control group (n = 82)	P value
Access site			＜0.001
Radial	19(38.1)	75(91.5)	
Femoral	0	1(1.2)	
Radial + femoral	23(61.9)	6(7.3)	
wire-crossing time ＞30 min	27(64.3)	31(37.8)	0.005
Retrograde approach	16(38.1)	6(7.3)	＜0.001
Number of stents	2.00 ± 1.2	1.74 ± 0.8	0.828
Procedure time, min	91.1 ± 25.4	69.4 ± 25.4	0.005
Contrast volume,mL	184.3 ± 91.6	158.1 ± 53.5	0.230
Procedure complications			
Coronary artery perforation	3(7.1)	1(1.2)	0.112
Coronary artery dissection/haematoma	5(11.9)	1(1.2)	0.017
Overall	8(19.0)	2(2.4)	0.003

Data presented as mean±standard deviation or number (%), Bold values highlight the p values under statistical significance (< 0.05)

### Determinants of PMI

In the univariate regression analysis, prior myocardial infarction ( odd ratio [OR], 2.812; 95% confidence interval [CI], 1.300-6.084; P = 0.009), previous PCI (OR, 2.688;95% CI, 1.251–5.773;P = 0.011), multivessel artery disease (OR, 5.150; 95% CI, 2.261– 11.735;P＜0.001), presence of calcification (OR, 3.753;95% CI, 1.704–8.264;P ＜0.001), retrograde approach (OR, 10.464; 95% CI, 3.737–29.297;P＜0.001), and presence of procedural complications (OR, 9.412; 95% CI, 1.889– 46.645;P  = 0.006) were associated with PMI development (Table 4). In the multivariate analysis, multivessel artery disease (OR, 4.347; 95% CI, 1.601– 11.809;P = 0.004), retrograde approach (OR, 4.036; 95% CI, 1.162–14.020; P = 0.028), and the presence of procedural complications (OR, 16.480; 95% CI, 2.515-107.987; P = 0.003) were predictors of PMI (Table 5).

**Table 4:** Univariate analysis of PMI

	P	or	95%CI
Age＞65years	0.585	1.236	0.577–2.650
Hyperlipidaemia	0.100	1.882	0.887–3.996
Diabetes	0.452	1.333	0.631–2.820
LVEF＜40%	0.114	2.002	0.846–4.737
Prior myocardial infarction	0.009	2.812	1.300-6.084
Prior stroke	0.688	1.166	0.551–2.468
Prior PCI	0.011	2.688	1.251–5.773
Multivessel artery disease^a^	P＜0.001	5.150	2.261–11.735
CTO length＞15 mm	0.415	1.463	0.585–3.657
Calcification	P＜0.001	3.753	1.704–8.264
Retrograde approach	P＜0.001	10.464	3.737–29.297
Procedure complications	0.006	9.412	1.899–46.645

Logistic binary regression; OR, odd ratio, CI, confidence interval; LVEF, left ventricular ejection fraction; PCI, percutaneous coronary intervention

^a^ Defined as ＞50% stenosis in 2 or more major coronary arteries

**Table 5:** Multivariate Logistic analysis of PMI

	P	or	95%CI
Prior myocardial infarction	0.247	3.674	0.406–33.282
Prior PCI	0.743	0.695	0.079–6.117
Multivessel artery disease^a^	0.004	4.347	1.601–11.809
Calcification	0.298	1.782	0.600-5.291
Retrograde approach	0.028	4.036	1.162–14.020
Procedure complications	0.003	16.480	2.515-107.987

^a^ Defined as＞50% stenosis in 2 or more major coronary arteries; OR, odd ratio; CI, confidence interval; LVEF, left ventricular ejection fraction; PCI, percutaneous coronary intervention

### Clinical outcomes

In this study, 12 patients (9.7%) developed MACE in hospital and during one month follow up. And there were two patients occurred (1.6%) all-cause death. Seven patients (5.6%) underwent target vessel revascularization, one of whom occurred acute myocardial infarction in hospital and the remaining six patients were in-stent thrombosis within one month follow up after procedure. Three patients (2.4%) occurred acute heart failure. In addition, patients with PMI had a higher incidence of MACE (19.0% vs 4.9%, P = 0.016) and target vessel revascularization (11.9% vs 2.4%, P ＜0.001) (Table 6).

**Table 6:** MACE between the two group in hospital and during one month follow up Number(%) M(P25,P75), mean ± SD

	PMI group(n = 42)	Control group (n = 82)	P value
Overall	8(19.0)	4(4.9)	0.016
All cause mortality	2(4.7)	0(0)	0.113
target vessel revascularization	5(11.9)	2(2.4)	＜0.001
Acute myocardial infarction	1 (2.3)	0 (0)	0.339
Acute heart failure	1(2.3)	2(2.4)	1.000

Data presented as mean±standard deviation or number (%); Bold values highlight the p values under statistical significance (<0.05)

## Discussion

The main findings of this study are: (1) The incidence of PMI is 33.9% after successful CTO-PCI; (2) PMI was predicted by the presence of multivessel artery disease, the use of retrograde approach and the occurrence of procedural complications; (3) Patients who develop PMI tend to have more MACE than those who do not develop PMI.

Back in 2015, the EXPLORE (Evaluating Xience and Left Ventricular Function in Percutaneous Coronary Intervention on Occlusion After ST-Elevation Myocardial Infarction) trial compared the effects of drug therapy alone versus direct PCI for CTO. The procedural success rate of direct PCI for CTO was only 71% [8]. Over recent years, with the application of advanced procedural techniques and evolution of procedural equipment, especially the adoption of retrograde approach, the overall procedural success rate of CTO-PCI has improved greatly. According to the 2019 European CTO Club statistics, the technical success rate of CTO has reached 90%, while the incidence of associated procedural complications has decreased to less than 5% [9]. With advances in the CTO-PCI technique, there is an increasing interest in procedure-related complications. Dautov et al. reported that the incidence of postprocedural PMI in 455 patients treated with CTO-PCI was approximately 30% (as measured by a postprocedural cTn elevation of more than 5 times the upper limit of the 99% reference value) [10], which is generally consistent with the incidence of PMI in our study (33.9%).

Although the pathogenesis of PMI has not been fully elucidated, some studies have suggested that collateral occlusion is the most common cause [11, 12]. Distal epicardial artery thrombosis also leads to slow/absent recurrent flow and PMI. [13] In addition, the use of techniques such as repeated balloon dilation, retrograde approach, and rotational atherectomy may also contribute to the occurrence of PMI [14]. In this study, the baseline data were comparable, and patients who developed PMI had a higher incidence of diabetes and dyslipidemia as well as a lower LVEF. This is largely consistent with previous studies on PMI [15,16]. Notably, patients in the PMI group were more likely to have prior PCI as well as coronary artery bypass graft (CABG) treatment. Study have shown that patients who were previously treated with CABG have more complex CTO lesions and a higher failure rate in antegrade wire escalation, often requiring retrograde access via bypass graft vessels. [17] Sioano et al. reported that approximately 10% of CABG patients will be treated with retrograde access via the graft vessel during CTO-PCI and these patients have a higher incidence of PMI [18]. With regard to angiographic characteristics, patients in the PMI group had more calcified lesions, had higher mean J-CTO scores, and were more likely to be treated with retrograde approach. This may be related to the more complex CTO lesions in the PMI group. Patients in the PMI group had a longer procedural time and a higher volume of contrast used. This also illustrates that the more complex procedural strategies used during CTO-PCI, the more procedural complications occurred. In this study, multivessel artery disease, the presence of procedural complications and the use of retrograde approach were predictors of PMI. According to a multicenter study of 26,000 patients, the incidence of coronary perforation during CTO-PCI was approximately 2% and the mortality of patients with perforation in 12 months was 5.8% which is double of patients without perforation [19]. In this study, only one patient who died from an intraoperative perforation during postprocedural hospitalization. Multivessel artery disease is defined as > 50% stenosis of 2 or more major coronary arteries. A meta-analysis demonstrated that multivessel artery disease not only increases the occurrence of MACE, it significantly impacts long-term patient prognosis [16]. The presence of blunt stumps and distal fibrous cap calcification make it more complex in antegrade CTO-PCI, as a result, an increasing number of patients require retrograde intervention [20] . However, the risk of PMI in retrograde intervention is 3 times higher than that of antegrade approach [21]. During retrograde CTO-PCI, the occlusion of collateral vessels by microcatheters or balloons will increase the risk of myocardial injury [22]. However, retrograde access can greatly improve the procedural success rate for and facilitate the patient’s vascular revascularization [23]. In this study, patients in the PMI group had more complex lesions (more calcified lesions and higher J-CTO scores), which made them more susceptible to PMI when retrograde access was used. Lesion length, J-CTO score, and the presence of calcification were also predictors of the occurrence of PMI after CTO-PCI in other related studies [15, 22]. It is hypothesized that these factors increase procedural difficulty as well as the length of procedure, which in turn increases the probability of PMI. Another study showed that about 16.1% of patients who underwent CTO-PCI had coronary slow-flow and no-reflow phenomenon, and these patients were also more likely to develop PMI [24].

Although many studies have confirmed the association of elevated myocardial markers with the development of MACE after PCI, there are few of PMI prognosis after CTO-PCI. Lo et al. reported that approximately 8.6% of patients who developed PMI (defined as an increase in the CK-MB level of more than 3 times the upper limit of normal) after CTO-PCI. In this study, the incidence of MACE in patients with PMI was 1.5 times than the control group [25]. The European Society of Cardiology (ESC) expert consensus in 2021 adopted cTn elevation greater than 5 times the upper limit of the 99% reference value after PCI as the universal definition of PMI [3], increasing centers started to adopt this definition for PMI-related studies. Using elevated cTn as the definition of PMI, Graca-Santos et al. showed that the occurrence of PMI after CTO-PCI was associated with the occurrence of MACE within 1 year [26]. Similarly, a meta-analysis showed the same results that patients with PMI had a higher incidence of MACE [27]. In this study, we studied only the occurrence of MACE in hospital and during one month follow up. The results showed that patients in the PMI group had a higher risk of MACE in hospital and during one month follow up. This may be related to the presence of more procedural complications in the PMI group. Another research showed that severe dissection, hematoma and perforation affected the distal blood perfusion which increased the operation time as well as aggravated myocardial ischemia [16].

## Limitations

The present study also has some limitations. First, it was a small single-center observational study. In the analysis of patients’ postprocedural MACE, we studied only the occurrence of one month MACE and did not perform long-term follow-up. In summary, the conclusions drawn from this study require further confirmation in a large multicenter study.

## Conclusion

In this single-center study, the incidence of PMI after CTO-PCI was 33.9%. Multivessel disease, retrograde approach and the presence of procedural complications were independent predictors of PMI. Patients with PMI were more likely to develop MACE during one month than those without PMI.

## Data Availability

The datasets generated and analyzed during the current study are not publicly due to a further study of this area but are available from the corresponding on reasonable request.
